# Supplementary figures and images for: Assessing the diagnostic accuracy of biochemical, anthropometric, and combined indices for metabolic syndrome prediction in a cohort from Qatar Biobank
Source: PLoS One. 2025 Dec 30;20(12):e0339340. doi: 10.1371/journal.pone.0339340 (PMC12753079; doi:10.1371/journal.pone.0339340)

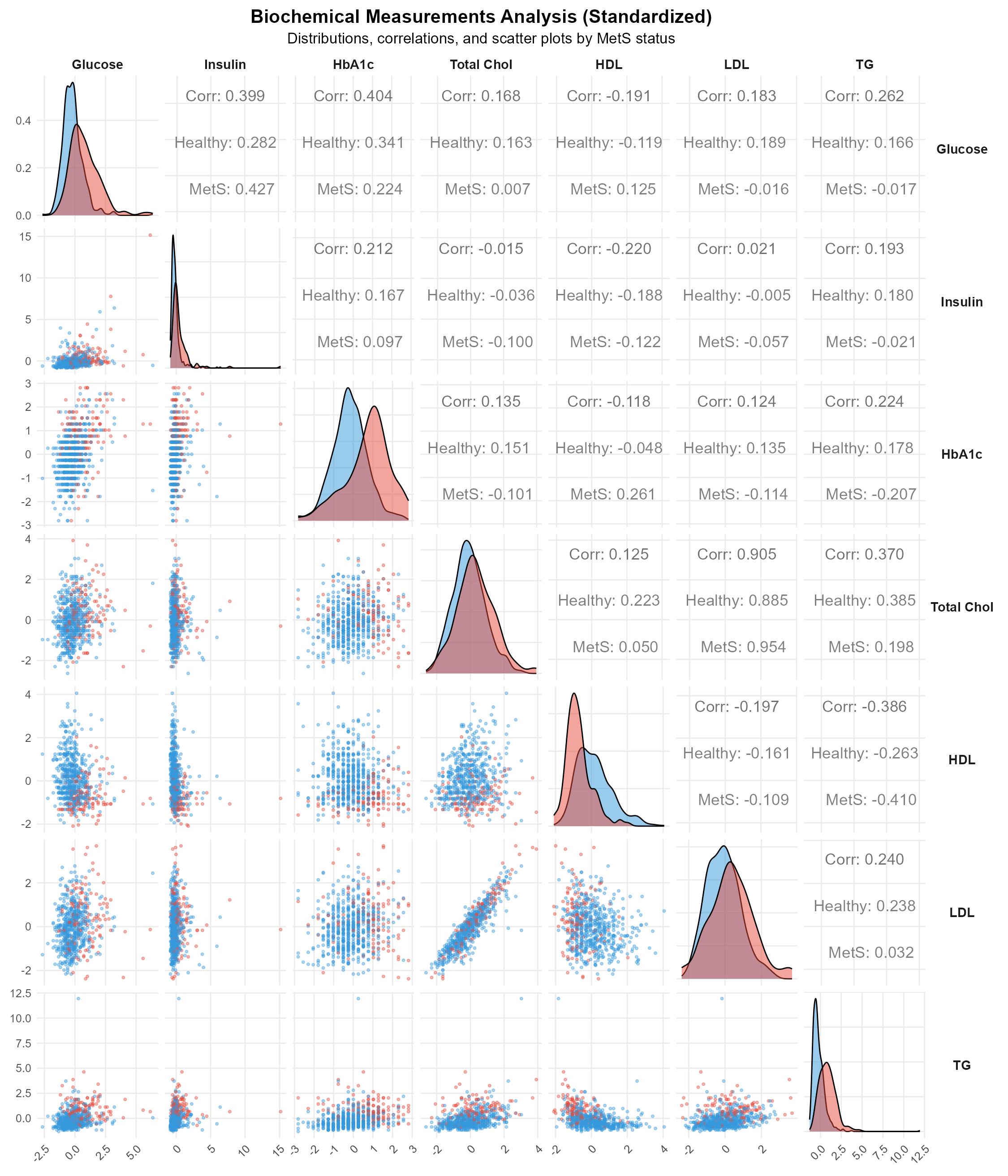

Supplement: S1 Fig — (TIF) [file pone.0339340.s001.tif]

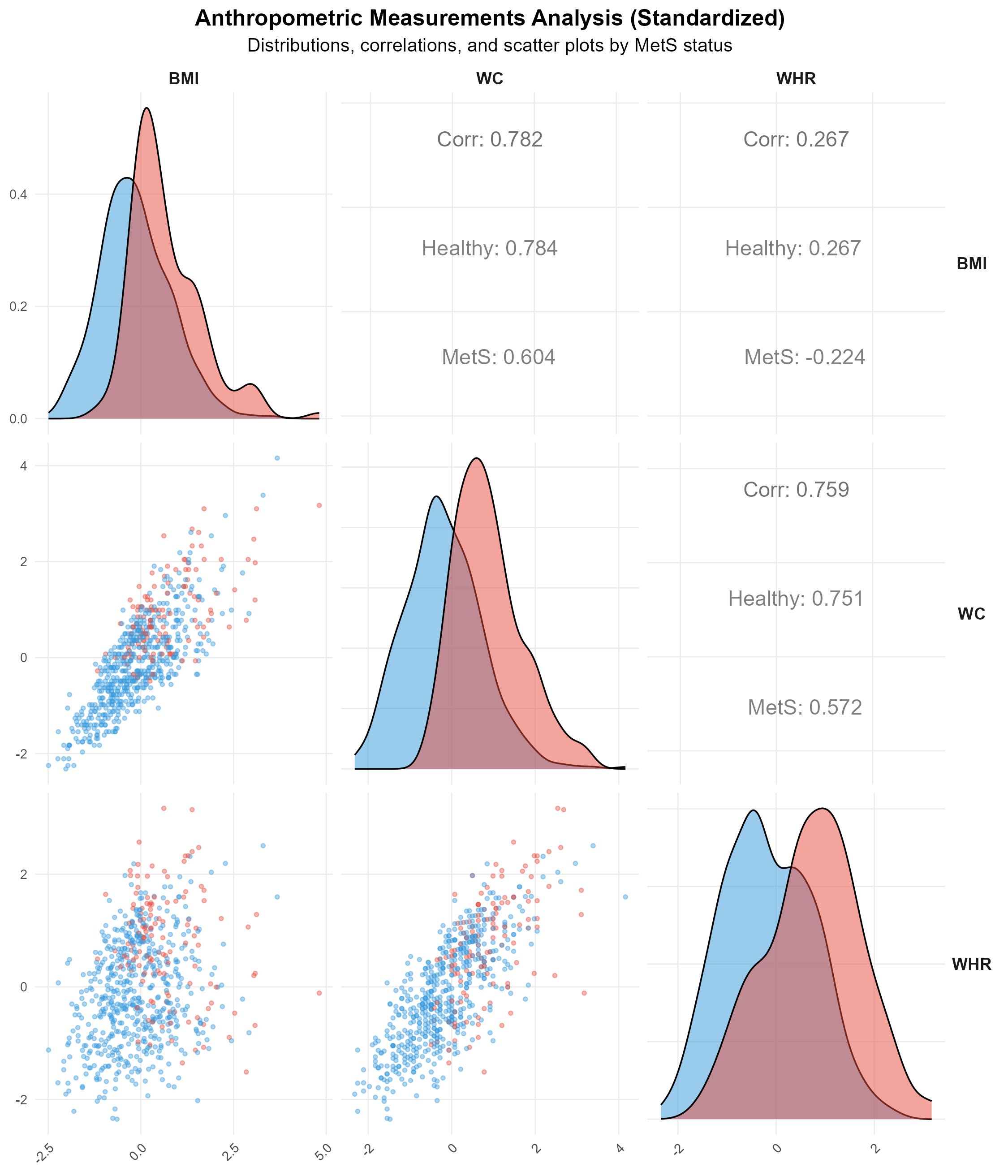

Supplement: S2 Fig — (TIF) [file pone.0339340.s002.tif]

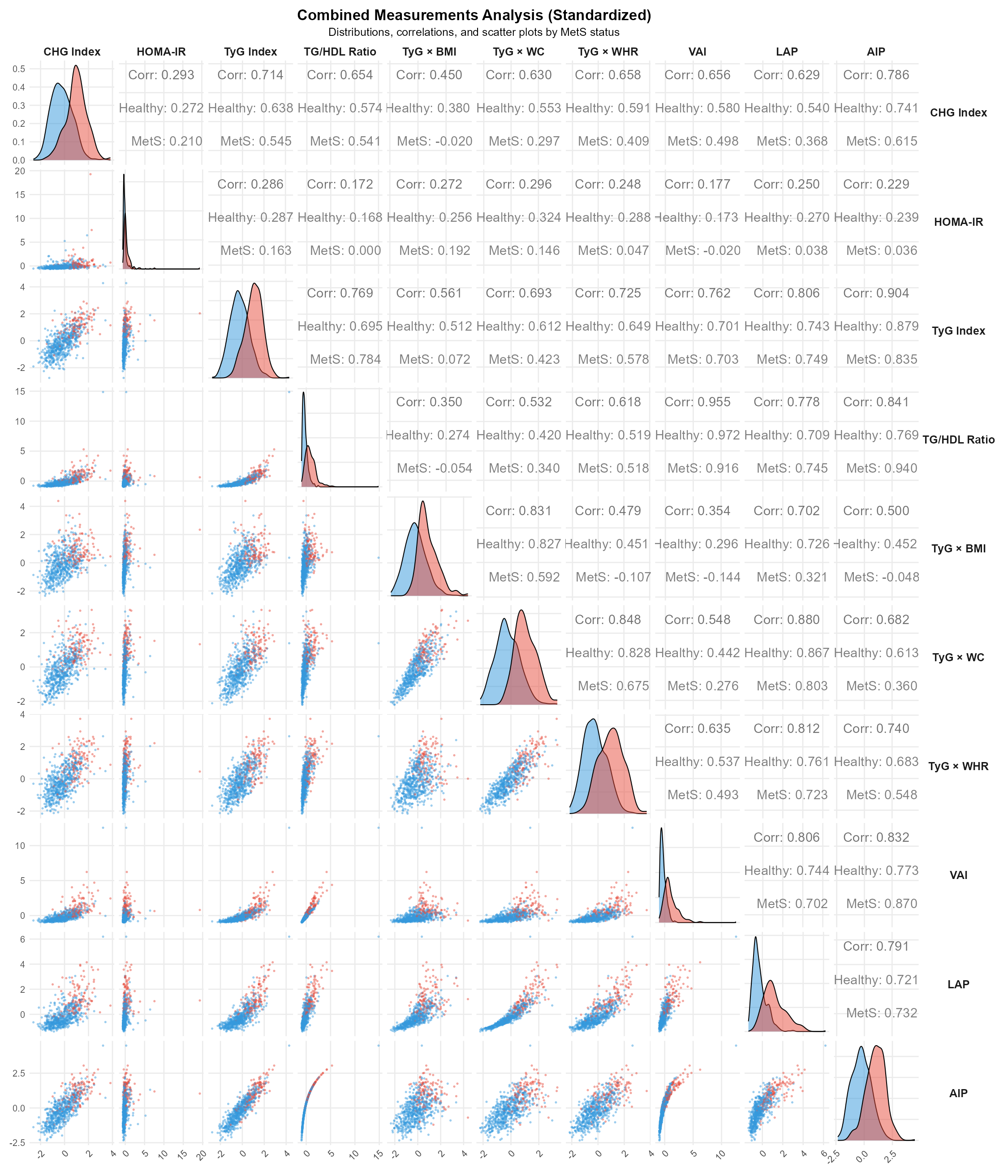

Supplement: S3 Fig — (TIF) [file pone.0339340.s003.tif]

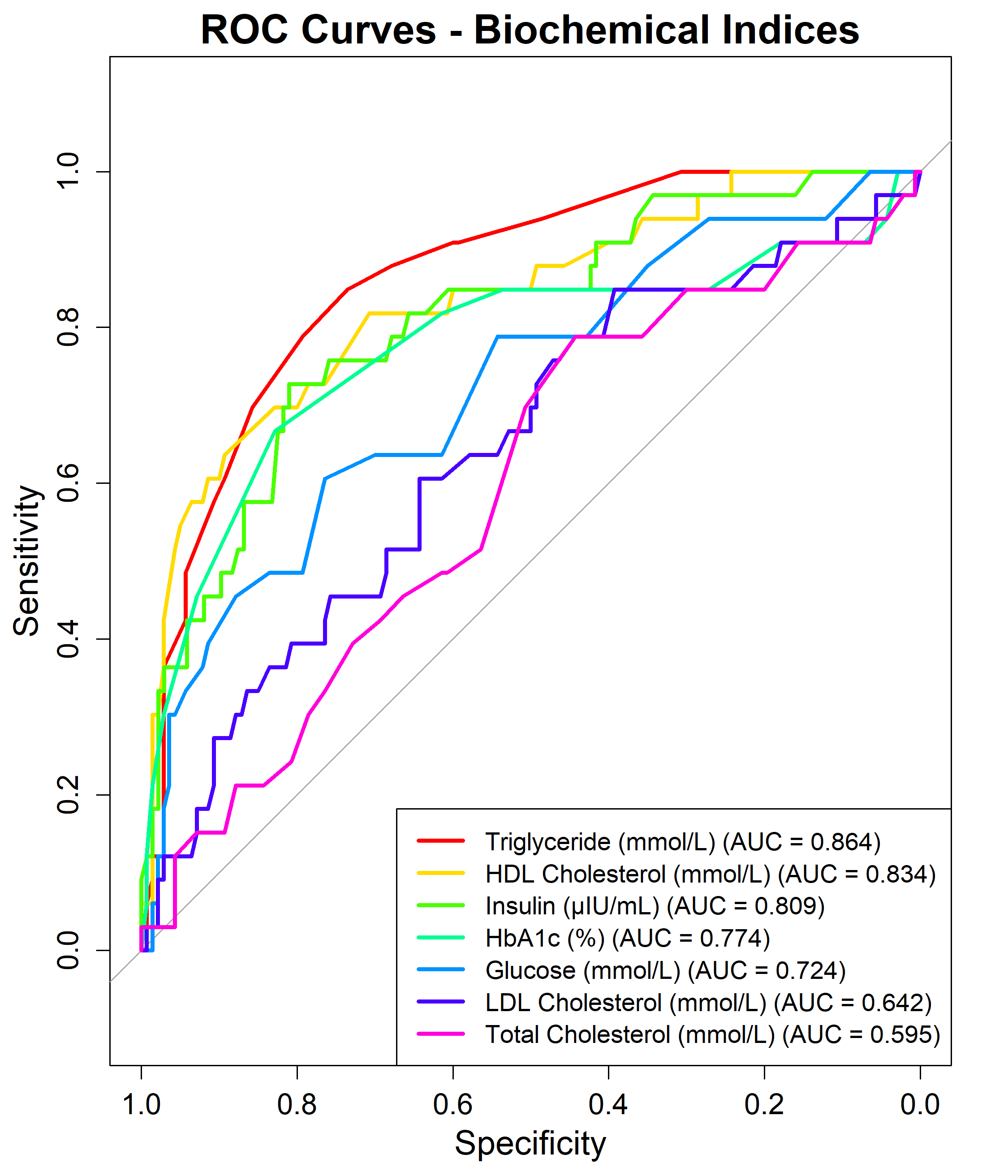

Supplement: S4 Fig — (TIF) [file pone.0339340.s004.tif]

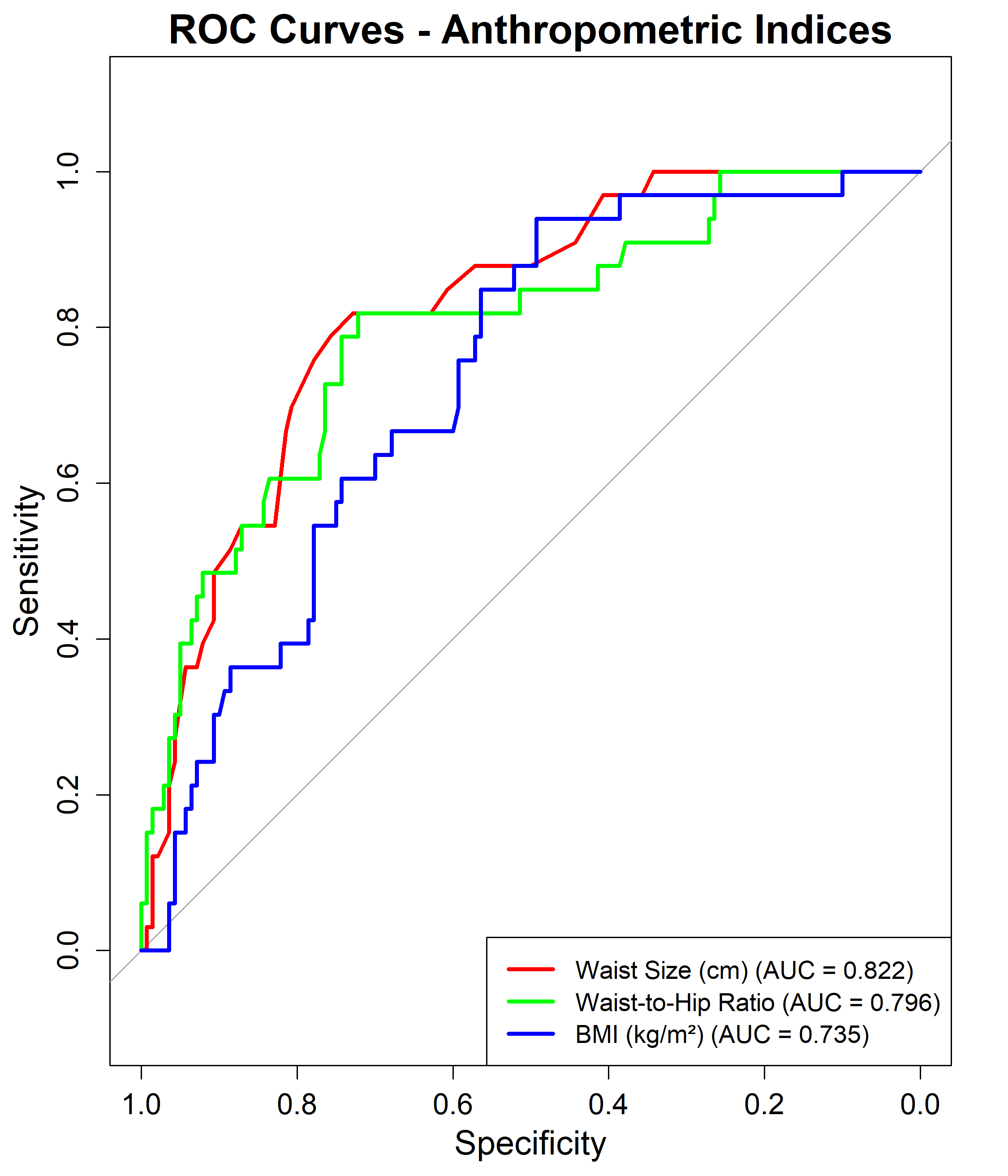

Supplement: S5 Fig — (TIF) [file pone.0339340.s005.tif]
