# Supplementary material for: Assessing the diagnostic accuracy of biochemical, anthropometric, and combined indices for metabolic syndrome prediction in a cohort from Qatar Biobank
Source: PLoS One. 2025 Dec 30;20(12):e0339340. doi: 10.1371/journal.pone.0339340 (PMC12753079; doi:10.1371/journal.pone.0339340)
Supplement: S1 Table — (DOCX) [file pone.0339340.s006.docx]

# **LAP (AUC = 0.896) Comparisons**

| Comparison | P-value |
| --- | --- |
| vs TyG×WC | 0.0010 |
| vs TyG×BMI | 0.0000 |
| vs Waist Size | 0.0000 |
| vs BMI | 0.0000 |
| vs WHR | 0.0000 |
| vs All basic measures | <0.0001 |

## **VAI (AUC = 0.877) Comparisons**

| Comparison | P-value |
| --- | --- |
| vs TyG×BMI | 0.0106 |
| vs Waist Size | 0.0052 |
| vs All traditional measures | <0.001 |
| vs AIP | 0.0028 |
| vs TG/HDL | 0.0033 |

## **TyG×WC (AUC = 0.872) Comparisons**

| Comparison | P-value |
| --- | --- |
| vs TyG×BMI | 0.0001 |
| vs TyG×WHR | 0.0081 |
| vs Traditional measures | <0.001 |

## **Traditional Measurements**

| Comparison | P-value |
| --- | --- |
| Waist Size vs BMI | 0.0000 |
| Waist Size vs WHR | 0.0016 |
| TG vs Glucose | 0.0146 |
| TG vs HDL | 0.0109 |
| Insulin vs HOMA-IR | 0.0000 |

## **Other Combined Indices**

| Comparison | P-value |
| --- | --- |
| TyG Index vs Traditional measures | <0.001 |
| TG/HDL vs BMI | 0.0000 |
| TG/HDL vs WHR | 0.0000 |
| TG/HDL vs Glucose | 0.0016 |
| AIP vs Traditional measures | <0.001 |

***Notes:*** Only comparisons with p < 0.05 are shown, P values are based on DeLong’s test for AUC comparison. Traditional measures include: BMI, WHR, Glucose, Insulin, HbA1c, HDL, TG
