# Supplementary material for: Assessing the diagnostic accuracy of biochemical, anthropometric, and combined indices for metabolic syndrome prediction in a cohort from Qatar Biobank
Source: PLoS One. 2025 Dec 30;20(12):e0339340. doi: 10.1371/journal.pone.0339340 (PMC12753079; doi:10.1371/journal.pone.0339340)
Supplement: Attachment — Submitted filename: PLOS_ONE_Rebuttal.docx [file pone.0339340.s008.docx]

**Point-by-Point Responses to the Editor and Reviewers’ Comments**

**EDITOR / JOURNAL REQUIREMENTS**

**1. Ensure manuscript meets PLOS ONE style requirements.**

Response: Manuscript re-formatted per PLOS ONE style: section headings, figure/table numbering, and file naming have been standardized.

**2 & 3. Data-availability compliance and justification.**

Response: The dataset is owned by Qatar Biobank (QBB) and contains identifiable participant data. Public release would breach MTA, NDA, and IRB terms and conditions.

Inserted statement:

“Data are available from Qatar Biobank under a controlled-access process and cannot be publicly shared due to IRB and data-transfer restrictions (IRB: Ex-2020-RES-ACC-0215-0125; Ex-2021-QF-QBB-RES-ACC-00049-0173). Requests should be directed to [qphi@qf.org.qa](mailto:qphi@qf.org.qa).”

**4. Funding statement placement.**

Response: The following text was retained only in the Funding Statement section and deleted from Acknowledgments:

The study was funded by the Qatar National Research Fund (Qatar Research Development and Innovation Council) [grant No. NPRP14S-0406-210150] and Qatar University [grants No. QUT2RP-CPH-24/25-477, QUST-1-CPH-2025-247, and QUST-2-CPH-2021-221]. M.A.Z. is supported by a Ph.D. graduate assistantship from the Office of Graduate Studies (Qatar University). The funders had no role in study design, data collection and analysis, decision to publish, or preparation of the manuscript.

**5. Abstract consistency.**

Response: Abstract on submission form and in manuscript now identical.

**6. Evaluation of suggested literature.**

Response: All reference papers were reviewed, and relevant ones cited in the Discussion.

**REVIEWER #1**

**1. Missing references in the Introduction.**

**Response:** We have now included supporting citations for all unreferenced claims.

**2. Introduction too long / restructure.**

Response: The section was condensed and reorganized for logical flow; redundant epidemiological detail was removed.

**3. Abbreviations not defined at first appearance.**

Response: Full manuscript checked—each abbreviation defined at first use.

**4. Medication data missing.**

Response: A lot of information on medication is missing in our dataset, so we could not, unfortunately, include this information in the analysis.

**5. Discussion lacks a comparable explanation (VAI).**

Response: We have included a comparative discussion. The inserted paragraph includes:

“The positive association between VAI and cardiometabolic disturbances observed in out cohort is consistent with the systematic review including 32 studies encompassing over 60,000 individuals across diverse age groups and regions, concluding that individuals with elevated VAI consistently exhibited higher blood pressure levels, independent of sex and age, underscoring VAI’s robustness as a marker of visceral adiposity and hemodynamic stress [49]. These findings reinforce our interpretation that VAI effectively captures the interplay between adipose tissue dysfunction and cardiometabolic risk. Complementary evidence from an Iranian population also highlighted the significance of VAI among adiposity indices. In their analysis of 9,704 adults from the MASHAD study, VAI remained significantly associated with hypertension in both men and women (OR = 1.03, 95% CI 1.02–1.04), even after adjustment for confounders [50]. Their findings reinforce the importance of VAI as a meaningful anthropometric indicator of blood pressure elevation and metabolic dysregulation across ethnic groups, supporting its inclusion in MetS risk assessment frameworks such as ours.”

**6. Add CHG index.**

Response: The CHG index is now incorporated into methods, analysis, and results tables. We added the following line in the Introduction:

“More recently Cholesterol, High-density lipoprotein, and Glucose (CHG) index have been used for the diagnosis of diabetes mellitus and is associated with MetS [19,20].”

**REVIEWER #2**

**1. Clarify that “predictive performance” refers to cross-sectional discrimination.**

Response: We replaced predictive-related terminology with “discriminatory ability/performance” throughout.

**2. Representativeness of Qatar Biobank sample.**

Response: We added a limitation paragraph discussing QBB volunteer bias and limited generalizability.

“A further limitation is the potential for selection bias. Participants in the Qatar Biobank are volunteers and may be healthier or more health-conscious than the general population. The cohort also predominantly comprises Qatari citizens, which may limit the generalizability of our findings to the large non-Qatari resident population in the country. Therefore, the prevalence and optimal thresholds of these indices should be validated in more diverse and representative community-based samples from Qatar and the wider Gulf region.”

**3. Clinical applicability of LAP**

Response: We added a discussion paragraph as follows:

“The clinical implementation of LAP is highly feasible, particularly in primary care settings. LAP requires only two common measurements: waist circumference, which is a simple anthropometric measure, and a fasting triglyceride level, which is part of a standard lipid panel. Given its simple calculation and high diagnostic accuracy demonstrated in our study, LAP and VAI represent a cost-effective and powerful tool that can be easily integrated into routine health check-ups in Qatar to improve early detection of MetS.”

**4. Age-stratified results.**

Response: We added an explanation for stronger performance in younger participants as follows:

“A particularly noteworthy finding from our study was the significantly higher discriminatory ability of all indices in individuals younger than 45 years. This age-related difference may be attributable to several factors. In older adults (≥45 years), the metabolic landscape is often complicated by a higher prevalence of comorbidities and the use of medications (e.g., statins, antihypertensives), which can alter lipid and glucose levels, thereby reducing the diagnostic clarity of these indices. Furthermore, physiological changes associated with aging, such as alterations in body composition and fat distribution, might weaken the association between these surrogate markers and MetS. This finding suggests that while these indices are exceptionally useful for identifying at-risk younger adults, their interpretation in older populations may require greater clinical nuance and consideration of confounding factors.”

**5. Data-availability revision.**

Response: We updated the statement as follows:

“Data are available from Qatar Biobank under a controlled-access process and cannot be publicly shared due to IRB and data-transfer restrictions (IRB: Ex-2020-RES-ACC-0215-0125; Ex-2021-QF-QBB-RES-ACC-00049-0173). Requests should be directed to [qphi@qf.org.qa](mailto:qphi@qf.org.qa).”

**6. Typographical and redundancy corrections.**

Response: All typos have been corrected, and redundant statements have been removed.

**7. Continuous variables standardized.**

Response: We added the following statement to the Methods: “For the logistic regression analysis, all continuous variables were standardized (converted to z-scores) to allow for the comparison of odds ratios across different measures.”

**8. Simplify tables / clarify labels.**

Response: We included abbreviation footnotes and uniform labeling for all tables.

**REVIEWER #3**

**1. Figures unclear/blurry.**

Response: We replaced those figures with high-resolution versions in corrected aspect ratios. All relevant information is also presented in the associated tables.

**2. Typographical errors.**

Response: We proofread the entire text; all typographical issues were now corrected.
