# Supplementary material for: Assessing the diagnostic accuracy of biochemical, anthropometric, and combined indices for metabolic syndrome prediction in a cohort from Qatar Biobank
Source: PLoS One. 2025 Dec 30;20(12):e0339340. doi: 10.1371/journal.pone.0339340 (PMC12753079; doi:10.1371/journal.pone.0339340)
Supplement: Attachment — Submitted filename: PLOS_ONE_Rebuttal_auresp_2.docx [file pone.0339340.s009.docx]

**Point-by-Point Responses to the Reviewers’ Comments**

**REVIEWER #3**

**1. The figures are hard to read and correlate to the note written. Can the figures be made more readable? Also, why not have the figures where the legend is located just like the tables? That would make the reading flow better even for reviewers.**

**Response:** We thank the reviewer for their feedback. We have addressed the two points as follows:

- **Figure Readability:** This issue has been fully resolved. We have replaced all figures with high-resolution versions, ensuring each one complies strictly with the journal’s technical specifications, including a minimum resolution of 300 DPI and adherence to the maximum dimensions of 7.5 x 8.25 inches. The revised figures are now crisp, clear, and fully legible.
- **Figure Placement:** Regarding the suggestion to embed figures within the text alongside their captions, this request is in direct conflict with PLOS ONE's mandatory author guidelines. The journal's formatting policy explicitly requires authors to submit figure files separately and **not** to embed them in the manuscript file. The guidelines state that captions must be listed in the manuscript text after the first paragraph of their citation.

Accordingly, we have strictly adhered to the journal’s submission protocol. To do otherwise would be a direct violation of the submission requirements. We are confident that with the vastly improved resolution, correlating the figures with their corresponding captions in the text is now straightforward. We trust this clarifies why we could not accommodate this specific suggestion and confirms our compliance with the journal's formatting standards.
